# Beyond inhaled medications: precision medicine and biologic therapies targeting the IL-33/TSLP/type 2 axis in COPD

**DOI:** 10.3389/fimmu.2026.1772031

**Published:** 2026-02-05

**Authors:** Guo-qiang Song, Bangsheng Chen, Tian-li He, Guo-qiang Hu

**Affiliations:** 1 Department of Respiratory Medicine, Changxing County Hospital of Traditional Chinese Medicine, Huzhou, Zhejiang, China; 2 Department of Emergency Medical Center, Ningbo Yinzhou No.2 Hospital, Ningbo, Zhejiang, China; 3 Department of Radiotherapy, Changxing County People’s Hospital, Huzhou, Zhejiang, China

**Keywords:** biologics, chronic obstructive pulmonary disease, IL-33, IL-4 receptor, IL-5, JKN2401, ST2, tezepelumab

## Abstract

Chronic obstructive pulmonary disease (COPD) is characterized by persistent airflow limitation and chronic airway inflammation, traditionally managed with inhaled bronchodilators and corticosteroids. However, a significant subset of patients exhibits suboptimal response to these inhaled therapies, and disease progression remains challenging to control effectively. Recent advances in understanding the inflammatory pathways underlying COPD have led to the development of biologic agents targeting critical cytokines and their receptors, including IL-4 receptor (IL-4R), IL-5, IL-5 receptor (IL-5R), IL-33, ST2, and thymic stromal lymphopoietin (TSLP). Emerging drugs such as JKN2401, TQC2731, and tezepelumab demonstrate promising therapeutic potential by modulating these specific inflammatory mediators. This review comprehensively summarizes the pathophysiological roles of these cytokines in COPD, the current progress in biologic drug development targeting these molecules, and the outcomes of recent clinical trials. By elucidating these novel therapeutic avenues, the article aims to provide a theoretical foundation and clinical guidance for precision medicine approaches in COPD management beyond conventional inhaled treatments.

## 1 Introduction

Chronic obstructive pulmonary disease (COPD) is a globally prevalent and debilitating respiratory condition characterized by persistent airflow limitation, chronic airway inflammation, and progressive destruction of lung parenchyma. Its incidence increases with age (1, 2). The pathophysiology of COPD is complex and multifactorial, predominantly driven by chronic exposure to noxious particles such as cigarette smoke and environmental pollutants, which induce oxidative stress and persistent inflammatory responses within the airways and lung tissue (1, 3). This chronic inflammation involves a dysregulated immune response with recruitment and activation of various innate and adaptive immune cells, including neutrophils, macrophages, dendritic cells, and lymphocytes, which collectively contribute to airway remodeling, mucus hypersecretion, and emphysematous changes (1, 4). Notably, immune dysregulation in COPD leads to impaired immune cell function and reduced host defense, predisposing patients to recurrent infections and exacerbations that accelerate disease progression (1).

Crucially, recent studies have further elucidated that the pathological impact of Type 2 inflammation extends beyond immune cell recruitment; upstream alarmins such as IL-33 and thymic stromal lymphopoietin (TSLP), along with related pathways like TL1A/DR3, act as potent drivers of airway remodeling, mucus hypersecretion, and epithelial-mesenchymal transition (EMT) (5, 6).Traditional pharmacologic management of COPD primarily relies on inhaled therapies, including long-acting bronchodilators such as long-acting beta-2 agonists (LABA) and long-acting muscarinic antagonists (LAMA), often combined with inhaled corticosteroids (ICS) in selected patients (7, 8). These treatments aim to relieve symptoms, improve lung function, reduce exacerbation frequency, and enhance quality of life. However, despite widespread use, inhaled therapies remain the main treatment for COPD acute exacerbations (9, 10). Moreover, the use of ICS is associated with potential adverse effects such as increased pneumonia risk (11, 12).

Recent advances in the understanding of COPD immunopathology have highlighted the heterogeneity of inflammatory phenotypes, including the identification of type 2(T2) (eosinophilic) inflammation in a subset of patients, which may represent a distinct treatable trait responsive to corticosteroids (13, 14). Beyond eosinophilic pathways, emerging evidence implicates immune dysregulation and inflammatory cell changes in COPD pathogenesis (1). These molecules, traditionally studied in asthma and other **T2** inflammatory diseases, are gaining attention for their roles in COPD, particularly in phenotypes with overlapping features or heightened eosinophilic activity (13, 14).

The limitations of current inhaled therapies and the recognition of distinct inflammatory endotypes have spurred interest in novel biologic agents targeting these specific immune pathways. Biologics such as monoclonal antibodies against IL-4Rα (e.g., dupilumab) are under active investigation for COPD treatment, with several agents having progressed to clinical trials (14). These studies focus on COPD burden with T2 inflammation and associations with eosinophil counts and corticosteroid response, without direct evidence on targeted therapies modulating immune responses or improving lung function beyond inhaled treatments (13, 14). Importantly, the identification of biomarkers such as blood and sputum eosinophil counts aids in patient stratification and therapeutic decision-making, enabling precision medicine approaches (15, 16).

This review aims to systematically summarize the biological functions of IL-4R, IL-5, IL-5R, IL-33, ST2, and TSLP in COPD pathogenesis, delineate the current landscape of biologic drug development targeting these pathways, and evaluate their potential clinical applications. By focusing on these emerging therapeutic targets beyond conventional inhaled medications, we seek to provide a comprehensive overview of advances in COPD treatment and highlight the translational potential of immunomodulatory biologics in addressing unmet clinical needs.

## 2 Immune inflammatory mechanisms of COPD and theoretical basis of biologic therapy

### 2.1 Characteristics of airway inflammation and cytokine networks in COPD

**Figure 1:**
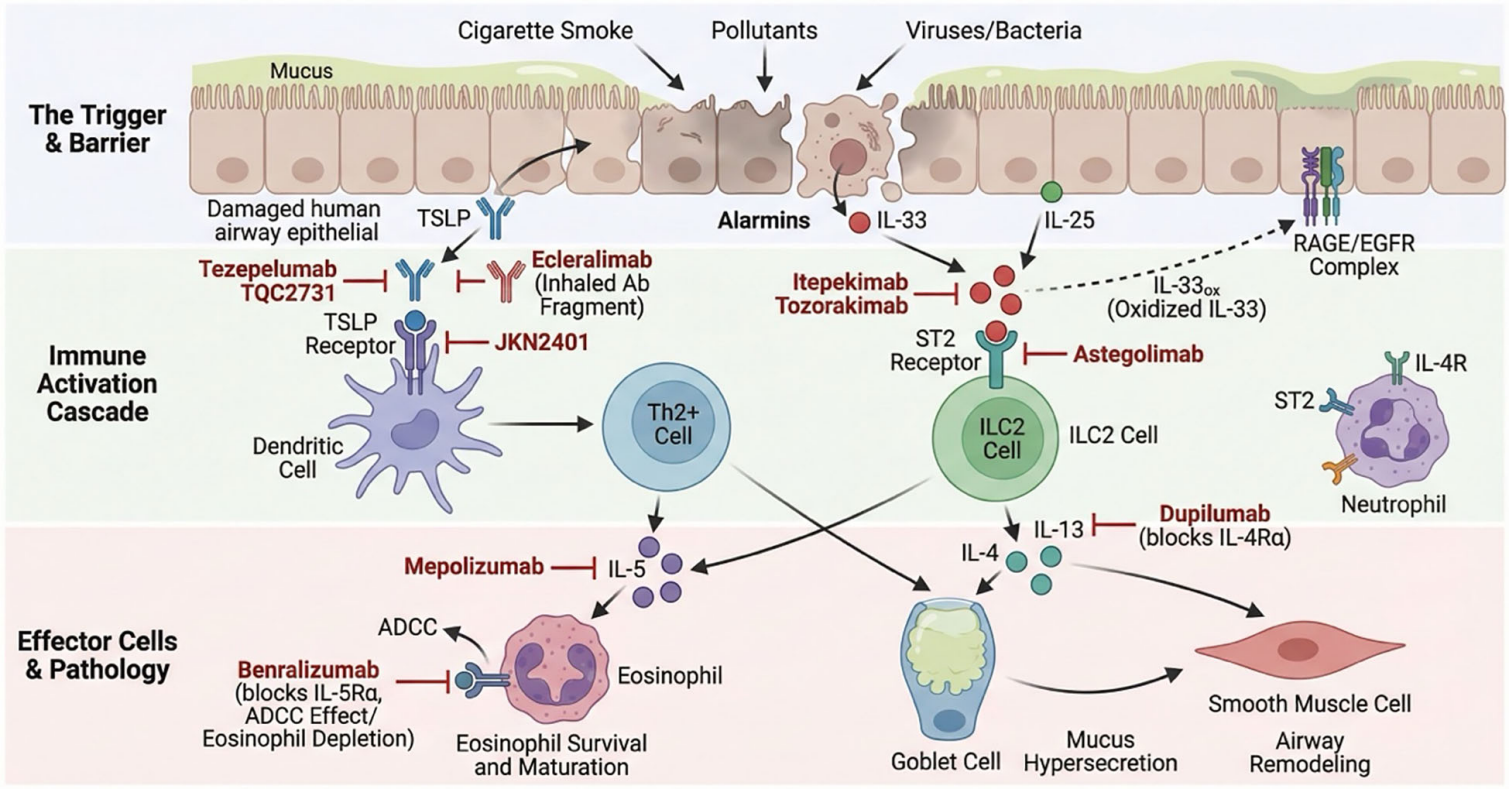
Pathophysiological mechanisms and therapeutic targets of T2-high and alarmin-driven COPD. This diagram illustrates the cascade of immune dysregulation and the sites of action for targeted biologic therapies in COPD (1). Initiation: Environmental insults (cigarette smoke, pollutants, viruses) damage the airway epithelial barrier, triggering the release of upstream alarmins: TSLP, IL-33, and IL-25. Notably, oxidized IL-33 (IL-33ox) may also signal through the RAGE/EGFR complex to directly promote epithelial remodeling independent of ST2 (2). Immune Activation: TSLP binds to its receptor on dendritic cells to prime Th2 differentiation. IL-33 binds to the ST2 receptor on Type 2 Innate Lymphoid Cells (ILC2s) and other effector cells. These events amplify the release of downstream type 2 cytokines (IL-4, IL-5, IL-13) (3). Effector Phase & Pathology: IL-5 is critical for eosinophil maturation, survival, and recruitment. IL-4 and IL-13 signaling via IL-4R drives goblet cell hyperplasia, mucus hypersecretion, and airway remodeling (4). Biologic Interventions: The figure depicts the specific targets of emerging and approved biologics: Tezepelumab and TQC2731 (anti-TSLP); JKN2401 (anti-TSLP Receptor); Itepekimab and Tozorakimab (anti-IL-33); Astegolimab (anti-ST2); Dupilumab (blocks IL-4Rα to inhibit IL-4/IL-13); Mepolizumab (anti-IL-5); and Benralizumab (anti-IL-5Rα, inducing eosinophil depletion via ADCC). TSLP, Thymic Stromal Lymphopoietin;IL-33, Interleukin-33; IL-25, Interleukin-25; IL-33ox, Oxidized Interleukin-33; IL-4/5/13, Interleukin-4, 5, or 13; ST2, Suppression of Tumorigenicity 2 (IL-33 Receptor); TSLPR, Thymic Stromal Lymphopoietin Receptor; IL-4R/IL-4Rα, Interleukin-4 Receptor/alpha subunit;IL-5Rα, Interleukin-5 Receptor alpha subunit; RAGE, Receptor for Advanced Glycation End-products; EGFR, Epidermal Growth Factor Receptor; Th2 Cell, Type 2 Helper T cell; ILC2, Type 2 Innate Lymphoid Cell; DC, Dendritic Cell; ADCC, Antibody-Dependent Cellular Cytotoxicity; Ab, Antibody.

COPD is primarily characterized by persistent airway inflammation that continues even after cessation of cigarette smoking, a major etiological factor. The inflammatory milieu in COPD airways is predominantly neutrophilic, with neutrophils playing a central role in tissue damage and disease progression (**Figure 1**). Neutrophil extracellular traps (NETs), which consist of DNA decorated with proteases and other inflammatory mediators, are significantly elevated in COPD patients and correlate with disease severity. These NETs promote airway epithelial cell proliferation and activation of the NF-κB pathway, leading to production of proinflammatory cytokines and dendritic cell maturation, thereby sustaining chronic inflammation; however, their role in type I interferon production and its link to chronic inflammation is less clear (17). This neutrophil-driven inflammation is complemented by the involvement of other innate immune cells such as macrophages and adaptive immune cells including T lymphocytes, which collectively perpetuate the inflammatory state (18, 19).

Although neutrophilic inflammation is dominant, a subset of COPD patients exhibits an eosinophilic inflammatory phenotype characterized by increased eosinophil counts in the airways. This eosinophilic phenotype is associated with elevated expression of T2 cytokines such as interleukin-4 (IL-4) and interleukin-5 (IL-5), which are classical mediators of T2 inflammation. IL-4 and IL-5 contribute to airway remodeling in allergic airway inflammation, though specific mechanisms such as mucus hypersecretion and smooth muscle hypertrophy require further clarification (20). The heterogeneity of inflammatory phenotypes in COPD suggests that differential cytokine profiles underlie distinct pathophysiological mechanisms, which may explain variable responses to therapies targeting inflammation.

Upstream regulators of airway inflammation include alarmin cytokines such as interleukin-33 (IL-33), TSLP, and the IL-33 receptor ST2. These epithelial-derived cytokines act as immune modulators involved in chronic airway inflammation, though specific activation of innate lymphoid cells, mast cells, and Th2 lymphocytes require further elucidation (21, 22). IL-33 is secreted via a nonclassical exosome trafficking pathway regulated by neutral sphingomyelinase 2, which is upregulated in COPD airway epithelium (23). The IL-33/ST2 axis has been implicated in promoting autoantibody production against airway epithelial components, linking airway microbiota dysbiosis to autoimmune responses that exacerbate COPD pathology (24). TSLP is detectable in COPD airway epithelial cells and is associated with altered secretion of inflammatory mediators, suggesting its potential as a therapeutic target (25).

The interplay between neutrophilic and eosinophilic inflammation is further modulated by cytokines such as IL-36 and IL-23, which are associated with neutrophilic airway inflammation and antimicrobial defense. Reduced levels of IL-36 in eosinophilic phenotypes may predispose patients to airway infections, highlighting the complex cytokine network regulating airway immune responses in COPD (26). Moreover, factors such as resistin-like molecule β (RELMβ) and extracellular heat shock protein 70 (eHsp70) have been shown to augment airway inflammation through pathways involving autophagy and NF-κB/MAPK signaling, respectively, contributing to the chronic inflammatory environment (27, 28).

The airway epithelium itself plays a crucial role as both a physical barrier and an active immunological organ. Cigarette smoke and other environmental insults impair epithelial barrier integrity by downregulating junctional proteins like E-cadherin and zonula occludens-1, leading to increased permeability and heightened inflammatory responses (29). In addition, epithelial cells produce cytokines such as IL-17A, which acts in an autocrine manner to amplify mucus production and neutrophilic inflammation, further contributing to airway obstruction (30). The persistence of epithelial dysfunction and inflammatory memory in COPD patients underscores the chronicity of the disease and the difficulty in reversing airway damage (21).

In summary, COPD airway inflammation is a multifaceted process involving a complex cytokine network with predominant neutrophilic inflammation and a notable subset of eosinophilic inflammation mediated by T2 cytokines. Upstream alarmins such as IL-33, ST2, and TSLP act as master regulators of immune activation, promoting chronic inflammation and airway remodeling. The heterogeneity of inflammatory phenotypes and the interplay of diverse cytokines suggest that personalized therapeutic strategies targeting specific cytokine pathways may be necessary to effectively manage COPD inflammation and progression.

### 2.2 Advantages and challenges of biologic therapy

Biologic therapies represent a significant advancement in the treatment of COPD by offering targeted intervention against specific inflammatory pathways implicated in disease pathogenesis. Unlike traditional inhaled medications that broadly suppress inflammation and often lead to systemic side effects, biologics are designed to selectively inhibit cytokines or their receptors, such as IL-4R, IL-5, IL-5R, IL-33, ST2, and TSLP. This targeted specificity allows for modulation of discrete inflammatory mechanisms (31, 32). Consequently, biologics can be particularly beneficial for COPD patients who exhibit limited response to conventional inhaled therapies, such as those with eosinophilic or T2 inflammation endotypes, where traditional treatments fail to adequately control exacerbations or disease progression (33, 34).

One of the most notable advantages of biologics lies in their ability to address the heterogeneity of COPD by focusing on specific inflammatory pathways that drive disease in subpopulations. For instance, monoclonal antibodies targeting IL-5 and its receptor have been investigated in eosinophilic COPD patients, a subgroup characterized by elevated blood eosinophil counts and T2 inflammation, with mixed evidence regarding their efficacy in reducing exacerbations and improving lung function (35, 36). Similarly, agents targeting IL-4Rα, such as dupilumab, have shown significant reductions in exacerbation rates and improvements in forced expiratory volume in one second (FEV1) in biomarker-enriched populations (34, 35). Moreover, biologics targeting alarmins—epithelial-derived cytokines like IL-33 and TSLP—have shown mixed results in COPD, with limited or inconsistent benefits on lung function and exacerbation rates (34, 37). The precision of these therapies aligns well with the evolving concept of personalized medicine, aiming to tailor treatment based on individual inflammatory profiles and biomarker status.

Despite these advantages, several challenges remain in the clinical application of biologics for COPD. A primary obstacle is the significant heterogeneity of the disease, which complicates patient selection and limits the generalizability of trial results. COPD encompasses multiple endotypes with distinct inflammatory and pathological features, and no single cytokine or chemokine dominates the disease process universally (32). This necessitates robust biomarker identification and validation to effectively stratify patients who are most likely to benefit from specific biologic agents. However, the current lack of standardized, widely accessible biomarkers impedes the routine clinical use of biologics and may contribute to inconsistent efficacy outcomes observed in trials targeting non- T2 inflammatory pathways (33, 34). Additionally, the modest or non-significant effects of some biologics in non-eosinophilic COPD populations underscore the need for improved phenotyping and mechanistic understanding (34).

Another challenge is the relatively small target population eligible for biologic therapy in real-world settings. Studies indicate that only a minority of COPD patients meet the stringent inclusion criteria of randomized controlled trials evaluating biologics, often less than 10% of the general COPD population (38). This limited applicability raises concerns regarding cost-effectiveness and accessibility, especially considering the high expense associated with biologic treatments. Furthermore, long-term safety data and the potential for adverse effects remain areas requiring ongoing surveillance, as biologics modulate critical immune pathways (39). Finally, the integration of biologics into existing treatment paradigms, including their use in combination with inhaled therapies and during acute exacerbations, demands further clinical investigation to optimize therapeutic regimens and timing (40).

In summary, biologic therapies offer a promising, targeted approach to managing COPD by addressing specific inflammatory mechanisms and reducing systemic side effects. Their advantages are most pronounced in patients with **T2** inflammation and eosinophilic phenotypes unresponsive to conventional inhaled therapies. However, the challenges of patient heterogeneity, biomarker identification, limited eligible populations, and long-term safety considerations necessitate continued research and refinement of personalized treatment strategies. Expanding biomarker-driven clinical trials and real-world studies will be critical to overcoming these hurdles and fully realizing the potential of biologics in COPD management (33–35).

## 3 IL-4 receptor and advances in targeted drugs

### 3.1 IL-4R expression and function in COPD

Interleukin-4 receptor plays a pivotal role in mediating the signals of IL-4 and IL-13, two key cytokines involved in T2 inflammatory responses. In COPD, a subset of patients exhibits a T2 inflammatory endotype characterized by eosinophilic airway inflammation, which shares some pathogenic mechanisms with asthma. IL-4 and IL-13, through IL-4R engagement, promote B-cell class switching to IgE production, release of pro-inflammatory mediators, disruption of epithelial barriers, and tissue remodeling. These cytokines are involved in eosinophilic inflammation and airway remodeling in COPD, although the precise mechanisms driving goblet cell hyperplasia and mucus hypersecretion remain to be fully elucidated (41, 42). The expression of IL-4R on bronchial epithelial cells and immune cells facilitates these processes, making it a critical mediator in the pathophysiology of COPD with T2 inflammation.

Recent clinical trials have demonstrated that targeting IL-4R with monoclonal antibodies, such as dupilumab, can reduce acute exacerbations and improve lung function in patients with eosinophilic COPD. Dupilumab blocks the shared IL-4Rα subunit, thereby inhibiting both IL-4 and IL-13 signaling pathways. This inhibition leads to a reduction in eosinophil chemotaxis without broadly suppressing antiviral immune responses, as shown in bronchial epithelial cells from COPD patients; however, reduction in mucus hypersecretion was not observed (43, 44). The selective blockade of IL-4Rα decreases the expression of eotaxin-3 (CCL26), a chemokine critical for eosinophil recruitment, which may underlie the observed clinical benefits in reducing exacerbation frequency. Notably, IL-4Rα inhibition does not significantly affect mucin 5AC gene expression induced by IL-4/IL-13, suggesting that additional mechanisms contribute to mucus hypersecretion in COPD (43).

The presence of IL-4 and IL-13 in the airway milieu of COPD patients is supported by elevated cytokine levels detected in airway fluids, and their receptors are expressed not only on immune cells but also on structural cells such as epithelial cells. This widespread expression underscores the multifaceted role of IL-4R signaling in COPD pathogenesis, including promoting airway remodeling and hyperresponsiveness. IL-5 receptor alpha (CD125) expression on lung neutrophils and eosinophils has been observed in COPD and emphysema patients, suggesting involvement of T2 pathways in disease severity; however, the specific impact of IL-4 receptor expression and signaling on neutrophil function in this context requires further investigation (45). Given that neutrophils are predominant in COPD inflammation, their modulation via IL-4R pathways indicates a complex interplay between T2 inflammation and neutrophilic responses.

Systemic inflammation in COPD, especially in patients with comorbid obesity, shows elevated levels of several pro-inflammatory cytokines, although IL-4 levels may not differ significantly compared to non-obese COPD patients. The referenced study found no significant differences in IL-4 levels between COPD patients with and without obesity, and did not address the relative relevance of IL-4R-mediated pathways in airway versus systemic inflammation (46). Additionally, novel treatments such as halotherapy and ozone therapy in COPD models exert anti-inflammatory effects via modulation of oxidative stress and inflammasome activation; however, indirect interactions with IL-4/IL-13 signaling and synergy with IL-4R-targeted therapies are not established (47, 48).

The therapeutic impact of IL-4R blockade in COPD has been substantiated by large randomized clinical trials like the BOREAS study, showing that dupilumab reduces exacerbation rates and improves lung function regardless of emphysema status or baseline disease severity measured by indices such as the BODE score. These findings demonstrate efficacy of IL-4R-targeted biologics in COPD patients with elevated blood eosinophils, though broad applicability and integration into personalized treatment regimens require further validation (49, 50). IL-4R antagonists combined with conventional inhaled therapies have shown efficacy in reducing COPD exacerbations; however, evidence for enhanced clinical outcomes and emphasis on targeting multiple inflammatory pathways is limited (49).

In summary, IL-4R mediates critical functions in COPD pathogenesis by facilitating IL-4 and IL-13 signaling, which promote T2 inflammation, mucus hypersecretion, and airway remodeling. Targeting IL-4R with monoclonal antibodies offers a promising therapeutic avenue to reduce exacerbations and improve lung function in eosinophilic COPD. The expression of IL-4R on diverse immune and structural cells, including neutrophils, indicates a complex role in modulating airway inflammation and disease progression. Future research should aim to elucidate the precise mechanisms by which IL-4R influences COPD heterogeneity and to optimize patient selection for IL-4R-targeted therapies.

### 3.2 IL-4R-targeted biologics development

Interleukin-4 receptor alpha (IL-4Rα) has emerged as a pivotal therapeutic target in T2 inflammatory diseases, with dupilumab—a monoclonal antibody against IL-4Rα—being one of the most successful biologics approved for asthma and several other allergic conditions. Dupilumab blocks signaling induced by both IL-4 and IL-13 through the T2 IL-4 receptor, thereby downregulating the molecular pathways that drive T2 inflammation (51). This dual inhibition is particularly effective in controlling eosinophilic inflammation, IgE production, and tissue remodeling associated with allergic airway diseases. Given the overlapping immunopathology between asthma and COPD, especially in patients exhibiting eosinophilic inflammation, the potential utility of IL-4Rα antagonists has garnered increasing attention in COPD research.

Clinical trials have begun to explore IL-4Rα blockade in COPD populations, focusing on subgroups characterized by T2 inflammation markers such as elevated blood eosinophils. IL-4Rα inhibitors have demonstrated efficacy in severe eosinophilic asthma; however, clinical evidence supporting its efficacy specifically in COPD populations remains limited compared to severe asthma (52). Notably, patients with higher fractional exhaled nitric oxide (FeNO) levels, indicative of IL-13-driven inflammation, showed better clinical remission rates after switching to dupilumab following suboptimal responses to anti-IL-5/5R therapies. This reference reports IL-13-dominant endotypes responsive to IL-4Rα targeting in severe eosinophilic asthma, but the existence of such endotypes in COPD remains to be confirmed (52). Such findings underscore the heterogeneity of COPD and the necessity for biomarker-driven patient stratification in therapeutic decision-making.

Beyond clinical efficacy, the pharmacological properties and delivery methods of IL-4Rα antibodies are evolving to enhance patient convenience and treatment adherence. Recent formulation studies have demonstrated the feasibility of developing inhaled dry powder forms of anti-IL-4Rα antibodies using spray drying and spray freeze drying techniques. These formulations maintained antigen-binding capacity and biological activity after storage, offering a promising non-invasive administration route particularly advantageous for severe asthma (52). The inhalation route may also enable targeted delivery to the lung tissue, potentially reducing systemic exposure and adverse effects.

At the molecular and cellular levels, IL-4Rα expression is widespread among immune cell subsets, with distinct surface and intracellular pools that respond differently to biologic therapy. Studies using multicolor spectral flow cytometry have revealed that naive B cells harbor high levels of IL-4Rα both on the surface and intracellularly, whereas neutrophils predominantly contain intracellular IL-4Rα. Treatment with dupilumab induces internalization of the receptor-antibody complex, decreasing total IL-4Rα expression, yet a dormant intracellular pool remains unoccupied, which might be mobilized under certain conditions (53). This compartmentalization could influence therapeutic efficacy and resistance mechanisms, suggesting that future biologics might need to address both receptor pools for optimal inhibition.

IL-4Rα targeting has also been investigated in other inflammatory diseases with overlapping T2 immune pathways, such as atopic dermatitis and eosinophilic esophagitis, where dupilumab has shown remarkable efficacy (51). While COPD is pathophysiologically distinct, the shared involvement of IL-4 and IL-13 in airway inflammation provides a rationale for repurposing IL-4Rα blockers in COPD phenotypes marked by eosinophilic inflammation. However, it is important to recognize that COPD encompasses diverse endotypes, and IL-4Rα blockade may be most effective in patients with a type 2-high inflammatory profile.

In summary, IL-4Rα-targeted biologics, exemplified by dupilumab, represent a promising therapeutic avenue beyond asthma, extending into COPD management for patients with eosinophilic inflammation. Clinical evidence indicates that these agents can reduce exacerbations and corticosteroid dependence in selected COPD populations. Advances in inhaled formulations and a deeper understanding of IL-4Rα receptor biology may further optimize treatment strategies. Nevertheless, larger and more definitive clinical trials are needed to establish the full therapeutic potential and to delineate the COPD subgroups that will benefit most from IL-4Rα inhibition.

### 3.3 Key clinical studies and efficacy evaluation

The evaluation of IL-4 receptor inhibitors in COPD has gained significant attention due to the pivotal role of T2 inflammation in a subset of COPD patients characterized by eosinophilic airway inflammation. Dupilumab, a monoclonal antibody that blocks IL-4Rα and thereby inhibits both IL-4 and IL-13 signaling pathways, has emerged as a leading biologic agent in this domain. A comprehensive multi-criteria decision analysis covering 20 clinical trials with 9,294 COPD patients demonstrated that dupilumab showed the most robust efficacy among biologics targeting T2 inflammation, particularly in eosinophilic COPD phenotypes. This was evidenced by consistent reductions in acute exacerbation rates and improvements in forced expiratory volume in one second, supported by high-quality trial designs (34). Furthermore, a network meta-analysis focusing on biologics targeting T2 inflammation revealed that dupilumab significantly reduced the annualized rate of acute exacerbations by 0.44 events per year compared to placebo (95% CI: -0.77 to -0.10). It also decreased St. George’s Respiratory Questionnaire (SGRQ) total scores by 3.41 points (95% CI: -6.00 to -0.82), indicating improved health-related quality of life, and increased pre-bronchodilator FEV1 by 0.06 L (95% CI: 0.00 to 0.12) (54). These findings suggest that IL-4R inhibition can effectively mitigate the frequency of COPD exacerbations and enhance pulmonary function and patient-reported outcomes in biomarker-enriched populations.

In addition to dupilumab, other biologics targeting T2 inflammation, such as anti-IL-5 agents (mepolizumab and benralizumab), have been investigated for their clinical efficacy in COPD. Meta-analyses of randomized controlled trials (RCTs) encompassing 2,837 patients treated with anti-IL-5/IL-5R therapies showed a collective reduction in exacerbation risk compared to placebo (rate ratio 0.88; 95% CI: 0.80-0.97). Specifically, benralizumab demonstrated a borderline significant reduction in exacerbation risk (rate ratio 0.92; 95% CI: 0.85-1.00), while mepolizumab exhibited a trend toward lower exacerbation risk that did not reach statistical significance (rate ratio 0.90; 95% CI: 0.77-1.06) (44). High-dose regimens of these agents appeared to confer greater benefit, with reductions in moderate-to-severe exacerbations by approximately 12% and emergency department visits or hospitalizations by 33% in eosinophilic COPD patients (peripheral blood eosinophils ≥200 cells/μL) (55). Improvements in quality of life measures such as SGRQ and CAT scores were limited or non-significant with anti-IL-5 therapies, while dupilumab showed statistically significant but clinically limited benefits (56). This difference may relate to the broader immunomodulatory effects of IL-4R blockade compared to IL-5 pathway inhibition, which primarily targets eosinophil survival and activation.

Assessment of safety and tolerability profiles is crucial for the clinical application of biologics in COPD, given the chronic nature of the disease and the need for long-term treatment. Across multiple clinical trials and meta-analyses, IL-4R inhibitors like dupilumab have demonstrated favorable safety profiles, with adverse event rates comparable to placebo. Similarly, anti-IL-5 and anti-IL-5R agents exhibited acceptable tolerability without significant increases in serious adverse events (44, 54). The consistency of safety data supports the feasibility of incorporating these biologics into COPD management, particularly for patients with eosinophilic inflammation who are inadequately controlled by standard inhaled therapies. Selection of patients based on biomarkers such as blood eosinophil counts and fractional exhaled nitric oxide may help identify subgroups with differential therapeutic responses, though evidence for enhanced efficacy and safety remains to be fully established (56, 57).

Beyond IL-4R and IL-5 targeting agents, biologics directed at alarmins such as IL-33, ST2, and TSLP have been evaluated, albeit with mixed results. Agents like tozorakimab, itepekimab, astegolimab, and tezepelumab have shown potential in improving lung function in COPD populations, though statistically significant reductions in exacerbation rates have not been consistently demonstrated (34, 58). These findings underscore the complexity of COPD pathophysiology and the challenges in translating mechanistic insights into consistent clinical benefits. Moreover, biologics targeting non- T2 inflammatory pathways, including anti-TNF-α, anti-IL-1β, anti-IL-17A, and anti-IL-8 agents, have largely failed to demonstrate efficacy in COPD, often limited by small sample sizes, early-phase trial designs, and lack of biomarker-driven patient selection (34).

The integration of patient-reported outcome measures (PROMs) alongside objective clinical endpoints has been limited but is increasingly recognized as essential for comprehensive efficacy evaluation. Systematic reviews highlight that while most biologics improve PROMs related to respiratory symptom burden compared to baseline, data remain sparse, especially in real-world settings. Dupilumab stands out as the only biologic with proven efficacy in randomized controlled trials for both objective and subjective measures (59). However, real-world evidence is still emerging, with limited patient numbers and retrospective designs restricting definitive conclusions. This gap indicates the need for prospective studies incorporating PROMs and real-world data to better understand patient-centered benefits and adherence patterns.

In summary, IL-4R inhibitors such as dupilumab have demonstrated significant clinical efficacy in reducing acute exacerbations, improving lung function, and enhancing quality of life in eosinophilic COPD patients, supported by robust trial data and favorable safety profiles. Anti-IL-5/IL-5R therapies provide moderate benefits, particularly at higher doses, while biologics targeting alarmins and non- T2 pathways show limited or inconsistent efficacy. The evidence underscores the importance of biomarker-based patient stratification to optimize therapeutic outcomes. Future research should focus on large-scale, biomarker-driven clinical trials, long-term safety monitoring, and incorporation of PROMs and real-world evidence to refine the role of biologics in COPD management. Personalized medicine approaches integrating inflammatory endotyping hold promise to transform COPD treatment paradigms and improve patient outcomes.

## 4 Advances in targeted therapy of IL-5 and IL-5 receptor

### 4.1 IL-5/IL-5R in eosinophilic inflammation of COPD

Interleukin-5 is a pivotal cytokine that regulates the biology of eosinophils, primarily by promoting their differentiation, activation, and survival. IL-5 acts through its receptor, IL-5R, expressed predominantly on eosinophils, facilitating their proliferation in the bone marrow and recruitment to the airway tissues. This cytokine-receptor axis is central to the pathophysiology of eosinophilic airway inflammation, a phenotype increasingly recognized in a subset of COPD patients. Approximately 20-40% of COPD patients exhibit eosinophilic airway inflammation, which is characterized by elevated eosinophil counts in blood and sputum samples (41, 60). The enhanced presence of IL-5 in these patients correlates with the increased generation and persistence of eosinophils, contributing to airway remodeling and exacerbation propensity. It is also noteworthy that the inflammatory microenvironment facilitates remodeling through interactions with extracellular matrix components. Research by Zhang et al. revealed that Syndecan-1 (CD138) significantly amplifies airway remodeling by strengthening TGF-β1/Smad3 signaling, suggesting that targeting the interplay between inflammatory cytokines and matrix proteoglycans could offer new therapeutic angles for controlling fibrosis and smooth muscle hyperplasia (61).

Clinical studies have shown increased IL-5+ ILC2s in eosinophilic COPD airways, but direct associations between elevated blood eosinophils, IL-5 levels, disease severity, and exacerbation frequency remain unconfirmed (62, 63). This eosinophilic phenotype responds differently to anti-inflammatory therapies compared to the neutrophilic phenotype. For instance, monoclonal antibodies targeting IL-5 or IL-5Rα, such as mepolizumab and benralizumab, have shown efficacy in reducing exacerbation rates in COPD patients with elevated eosinophil counts. Meta-analyses indicate that anti-IL-5 therapies reduce exacerbation rates in eosinophilic COPD, although specific quantifications vary across studies (62, 64). These therapies also extend the time to first exacerbation and demonstrate a favorable safety profile. Current data regarding the effects of IL-5/IL-5R blockade on lung function or quality of life measures in COPD remain limited (61).

The pathobiological mechanism underlying IL-5’s role in eosinophilic COPD involves the activation of T2 innate lymphoid cells (ILC2s), which produce IL-5 and express IL-5 receptors. Studies have identified increased numbers of IL-5+ ILC2s in the sputum and peripheral blood of eosinophilic COPD patients compared to non-eosinophilic COPD and healthy controls. These ILC2s also express receptors for epithelial-derived alarmins such as TSLP, IL-33, and IL-25, which are released in response to cigarette smoke and other insults, further amplifying IL-5 production and eosinophilic inflammation (63). The correlation between sputum eosinophil percentages and ILC2 numbers supports the concept that epithelial alarmin-driven ILC2 activation sustains eosinophilic inflammation in COPD airways. This interplay suggests that IL-5 not only promotes eosinophil survival but also integrates signals from the airway epithelium to perpetuate T2 inflammation.

Interestingly, IL-5 receptor alpha expression has also been detected on lung neutrophils in COPD patients, indicating a broader role of IL-5 signaling beyond eosinophils. CD125 (IL-5Rα) expression on neutrophils in COPD suggests involvement of T2 pathways in inflammation and disease severity; however, direct contributions of T2 cytokines to neutrophilic inflammation and therapy responsiveness are not established (45). This finding extends the understanding of IL-5/IL-5R involvement in COPD pathogenesis and may partly explain the heterogeneity in treatment responses observed with IL-5-targeted biologics.

Overall, the elevation of IL-5 and its receptor in eosinophilic COPD patients underscores the importance of this axis in disease progression and exacerbation risk. Targeted therapies against IL-5/IL-5R have demonstrated clinical benefits in reducing exacerbations in biomarker-selected populations, highlighting the necessity of precise phenotyping for personalized treatment strategies. Given the complexity of IL-5 signaling involving multiple immune cell types and epithelial interactions, further research is warranted to delineate the full scope of its role and optimize therapeutic interventions (34).

### 4.2 Representative IL-5/IL-5R biologics

Interleukin-5 and its receptor play pivotal roles in eosinophil biology, including their maturation, activation, and survival, which are central to the pathogenesis of eosinophilic airway diseases such as severe asthma and certain phenotypes of COPD. Targeting IL-5/IL-5R with monoclonal antibodies has become a cornerstone in the management of eosinophilic inflammation, particularly for patients inadequately controlled by conventional therapies. Among the representative biologics, mepolizumab and benralizumab have gained widespread clinical use and regulatory approval, with expanding evidence supporting their efficacy and safety profiles.

Mepolizumab is a humanized monoclonal antibody that binds directly to IL-5, preventing its interaction with IL-5Rα on eosinophils, thereby reducing eosinophil levels in blood and tissue. Clinical trials and real-world studies have demonstrated that benralizumab significantly decreases exacerbation rates and oral corticosteroid (OCS) use in patients with severe eosinophilic asthma; evidence for mepolizumab in this context is less robust (39, 65). In COPD, mepolizumab has shown promise in reducing exacerbations in patients with elevated blood eosinophils, although the magnitude of benefit is more modest compared to asthma (55, 66). Mepolizumab has been evaluated for safety in eosinophilic airway diseases; although long-term safety data require further accumulation (36). Mepolizumab is an established treatment for EGPA and has shown efficacy in eosinophilic asthma with comorbid bronchiectasis; however, evidence from these references is limited to case reports and specific populations (67, 68).

Benralizumab, an afucosylated monoclonal antibody targeting IL-5Rα, induces antibody-dependent cell-mediated cytotoxicity leading to near-complete depletion of eosinophils and basophils. This mechanism results in rapid and sustained eosinophil depletion, which correlates with clinical efficacy. Benralizumab has demonstrated efficacy in severe eosinophilic asthma; however, the precise magnitude of reduction in exacerbation frequency varies across studies (38, 39). In COPD populations characterized by eosinophilic inflammation, benralizumab has shown trends toward reducing exacerbation risk, particularly in patients with blood eosinophil counts ≥300 cells/μL, though some studies report borderline statistical significance (44, 55). Benralizumab has been used successfully in cases of immunotherapy-induced eosinophilic complications; although evidence for its advantages in this specific subgroup remains limited (69).

Mepolizumab and benralizumab have been evaluated for safety and usage patterns, and direct head-to-head comparisons of efficacy are currently lacking (70, 71). Biomarker-guided approaches, including baseline blood eosinophil counts and FeNO, have been explored to predict treatment response; however, higher FeNO levels are associated with greater reduction in exacerbations but not necessarily with improved lung function or corticosteroid-sparing effects (72, 73). Switching between biologics is a common clinical practice in cases of partial or nonresponse, with evidence supporting potential benefit; however, evidence on safety of such strategies is limited (74, 75).

Emerging IL-5/IL-5R-targeted agents such as JKN2401 and TQC2731 are currently under development, aiming to enhance efficacy, dosing convenience, or safety profiles. Although detailed clinical data remain limited, these novel agents represent the ongoing evolution of biologic therapies targeting eosinophilic inflammation. Their development reflects the need to address heterogeneous patient responses and to optimize individualized treatment regimens in eosinophilic airway diseases.

In summary, mepolizumab and benralizumab constitute the principal IL-5/IL-5R biologics with established roles in managing eosinophilic asthma and subsets of COPD characterized by T2 inflammation. Their efficacy in reducing exacerbations, corticosteroid use, and improving quality of life is supported by robust clinical and real-world evidence. Biomarker-driven patient selection and therapeutic monitoring are critical to maximizing clinical benefits. The advent of new IL-5/IL-5R-targeted agents promises to further refine treatment options, although additional research is necessary to establish their place in clinical practice. These therapies underscore a paradigm shift towards precision medicine in chronic airway diseases, addressing unmet needs beyond inhaled medications (44, 55, 66, 70).

### 4.3 Clinical trial data and efficacy analysis

Interleukin-5 targeted therapies have emerged as promising biologics in the management of COPD particularly for patients exhibiting eosinophilic inflammation, a phenotype associated with increased exacerbation risk and responsiveness to anti-inflammatory treatment. Mepolizumab, an anti-IL-5 monoclonal antibody, has been extensively studied in this context. Clinical trials have investigated mepolizumab in COPD patients with elevated blood eosinophil counts (≥200 cells/μL), but conclusive evidence regarding exacerbation rate reduction remains to be solidified (36, 54). Real-world data further support these findings, showing a significant reduction in exacerbation frequency—up to a 2–3 fold decrease—and improvements in patient-reported outcomes such as COPD Assessment Test (CAT) scores after sustained anti-IL-5 therapy (66). However, it is noteworthy that some studies report a slight decline in absolute FEV1 values despite clinical benefits, suggesting that lung function parameters may not fully capture the therapeutic impact on disease activity and exacerbation control.

Beyond mepolizumab, benralizumab, an anti-IL-5 receptor α monoclonal antibody, has also shown efficacy in reducing moderate-to-severe exacerbations and improving quality of life scores in COPD patients with eosinophilic inflammation, as evidenced by network meta-analyses comparing multiple biologics targeting **T2** inflammation (54). Both agents exhibit favorable safety profiles, comparable to placebo, which is encouraging for their incorporation into clinical practice. The therapeutic benefits appear to be dose-dependent, with higher doses yielding more pronounced reductions in exacerbation rates and emergency department visits (55). These findings underscore the importance of appropriate dosing strategies tailored to patient-specific inflammatory profiles.

A critical aspect of optimizing biologic therapy in COPD involves the use of biomarkers to guide individualized treatment. Blood eosinophil counts have been the most widely studied biomarker for predicting response to IL-5 targeted therapies. Elevated peripheral blood eosinophil levels correlate with increased exacerbation risk; however, evidence for greater likelihood of benefiting from eosinophil-depleting biologics in COPD is limited (76, 77). However, eosinophil counts can be variable and influenced by multiple factors, and their predictive accuracy for sputum eosinophilia—a more direct measure of airway inflammation—is imperfect. Therefore, integrating additional biomarkers and clinical characteristics is essential to refine patient selection. Recent reviews advocate for a precision medicine approach in COPD, incorporating phenotypic and endotypic classification alongside biomarker profiles to identify treatable traits and optimize therapeutic outcomes (78, 79). This strategy holds promise for enhancing the efficacy of biologics by targeting those most likely to respond, thereby improving cost-effectiveness and patient care.

Emerging biologics targeting upstream epithelial alarmins such as IL-33 and TSLP are under investigation; early clinical data support IL-33 blockade reducing exacerbations in COPD, while clinical data for anti-TSLP therapies remain limited (80, 81). These agents may complement IL-5 targeted therapies by modulating diverse inflammatory pathways involved in COPD pathogenesis. Imaging endpoints assessed by advanced MRI techniques are gaining attention as sensitive measures to evaluate disease progression and may have potential in assessing biologic impact, though specific evidence for airway wall thickness and ventilation defects as surrogate markers for biologics is limited (31, 82).(see in **Table 1**).

**Table 1:** Efficacy of key biologic therapies in COPD clinical trials.

Drug	Target	Key trial(s)	Exacerbation reduction	FEV1 change	SGRQ change	Safety profile	References
Dupilumab	IL-4R	BOREAS, NOTUS	30-34% (MD -0.44 events/year; higher in high FeNO/eos ≥300 cells/μL) (34, 54)	+0.06 L (+82–83 mL; early improvement) (34, 54)	-3.41 points (clinically meaningful) (54)	No increase in SAEs; favorable	(34, 50, 54)
Mepolizumab	IL-5	METREX, METREO, MATINEE	18-23% (rate ratio 0.88 in eos ≥150/300 cells/μL; significant in MATINEE) (44, 55, 64),	Modest or N/A (pooled analysis shows eos-dependent) (36, 54)	Limited/non-significant (no difference) (56, 66),	Favorable, no SAE increase	(36, 44, 54, 55, 64, 66)
Benralizumab	IL-5R	GALATHEA, TERRANOVA	12-33% (MD -0.21 at 10 mg in eos ≥200 cells/μL; *post-hoc* ≥3 prior exac/triple therapy) (44, 55)	Improvement (*post-hoc* eos-dependent) (54)	-1.70 points (100 mg dose)	No SAE increase; favorable	(39, 44, 54, 55)
Tezepelumab	TSLP	COURSE (Phase 2a)	17% overall (significant in eos ≥150 cells/μL; pending Phase 3 like ALIENTO/ARNASA) (34, 58)	N/A (eos-dependent trends) (34)	N/A (34)	Favorable	(34, 58, 80, 81)
Itepekimab	IL-33	Phase 2 (NCT03546907)	Mixed (42% in ex-smokers/former smokers; not in current smokers) (34, 83)	Modest gains (not significant) (34, 37)	N/A (34)	No SAE increase	(34, 58, 81, 83)
Astegolimab	ST2	COPD-ST2OP (Phase 2a)	No significant overall (greater in low eos; trends in high eos) (84, 85)	Trends toward improvement (greater in high eos) (84)	Improved (greater in high eos) (84)	Favorable, no SAE increase	(34, 84, 85)
Tozorakimab	IL-33	Phase 2 (OBERON/PROSPERO)	Mixed (benefits in ≥2 prior exacerbations; effective in current/former smokers) (34, 58)	+24 mL (not significant) (34)	N/A (34)	Favorable	(34, 58)
JKN2401	TSLP-R	Preclinical/Phase I-II	N/A (early; potential TSLP modulation; ongoing trials) (86)	N/A (86)	N/A (86)	N/A (preliminary safe) (86)	(86)
TQC2731	TSLP	Phase II (ongoing, FRONTIER-4)	N/A (preliminary inhibitory effects; pending results) (86)	N/A (86)	N/A (86)	N/A (preliminary safe) (86)	(86)

COPD, Chronic Obstructive Pulmonary Disease; IL-4R, Interleukin-4 Receptor; IL-5, Interleukin-5; IL-5R, Interleukin-5 Receptor; IL-33, Interleukin-33; ST2, Suppression of Tumorigenicity 2; TSLP, Thymic Stromal Lymphopoietin; TSLP-R, Thymic Stromal Lymphopoietin Receptor; MD, Mean Difference; FeNO, Fractional Exhaled Nitric Oxide; eos, Eosinophils; exac, Exacerbations; FEV1, Forced Expiratory Volume in 1 Second; L, Liter; mL, Milliliter; SGRQ, St. George's Respiratory Questionnaire; SAEs, Serious Adverse Events.

In summary, IL-5 targeted biologics like mepolizumab and benralizumab have demonstrated efficacy in reducing COPD exacerbations and improving patient-reported outcomes in eosinophilic phenotypes, supported by both randomized controlled trials and real-world evidence. The integration of biomarkers, especially blood eosinophil counts, facilitates personalized treatment approaches, although further refinement is needed to enhance predictive accuracy. Ongoing research into alarmin-targeted therapies and novel imaging biomarkers is expanding the therapeutic landscape, aiming to address the heterogeneity of COPD and improve long-term disease management. It is anticipated that future large-scale, biomarker-driven clinical trials will clarify optimal patient selection and combination strategies to maximize the benefits of biologic therapies in COPD.

## 5 Research progress on the IL-33/ST2 axis and related biologics

### 5.1 Biological functions of the IL-33/ST2 signaling pathway

Interleukin-33, a member of the IL-1 cytokine family, functions primarily as an alarmin released by damaged or stressed cells, particularly epithelial and endothelial cells, to alert the immune system and initiate inflammatory responses. This cytokine is constitutively expressed in the nuclei of tissue-lining and structural cells, including airway epithelial cells and fibroblasts, and upon cellular injury or necrosis, IL-33 is rapidly released into the extracellular milieu where it exerts its biological effects (87, 88). Acting as an early danger signal, IL-33 binds to its specific receptor, suppression of tumorigenicity 2 (ST2, also known as IL1RL1), which exists in both membrane-bound (ST2L) and soluble (sST2) forms. IL-33 signals through its receptor ST2 and the distinct functional roles of membrane-bound versus soluble ST2 warrant further investigation (88, 89).

The IL-33/ST2 axis activates immune cells including mast cells, eosinophils, basophils, and ILC2s, promoting T2 immune responses; however, activation of Th2 lymphocytes, regulatory T cells, and production of IL-5, IL-9, and IL-13 are not yet fully characterized (90–92). This activation cascade is crucial in orchestrating inflammatory reactions in diverse pathological contexts, including allergic airway diseases, autoimmune disorders, and chronic inflammatory conditions. IL-33 induces IL-9 expression and modulates ILC2 proliferation and marker expression; direct evidence for proliferation and activation via ST2 contributing to amplification of T2 cytokine production and allergic inflammation is limited (92). IL-33/ST2 signaling modulates mast cell function by activating NF-κB and MAPK pathways; while its role in promoting pro-inflammatory mediator release requires further verification (90).

In the respiratory system, the IL-33/ST2 pathway is intimately involved in airway inflammation and remodeling. Elevated levels of IL-33 and ST2 have been detected in COPD patients; however, their direct correlation with disease severity remains to be consistently established (93, 94). IL-33 released from damaged airway epithelial cells binds to ST2 on various immune cells, leading to airway eosinophilia, hyperresponsiveness, and tissue remodeling characteristic of COPD pathology. Recent mechanistic studies have deepened our understanding of these structural changes. For instance, Zhang et al. successfully established a humanized IL-33 mouse model, demonstrating that human IL-33 directly promotes severe airway remodeling and Muc-5ac secretion, providing a translational link between alarmin release and mucus hypersecretion (5). Furthermore, the complexity of the Type 2 axis is highlighted by the identification of the TL1A/DR3 axis—a member of the TNF superfamily that functions synergistically with Type 2 inflammation—which has been shown to augment EMT in airway epithelial cells, thereby accelerating structural airway obstruction (95). Interestingly, IL-33 can also engage in ST2-independent signaling pathways, such as forming complexes with the receptor for advanced glycation end products (RAGE) and epidermal growth factor receptor (EGFR) on airway epithelial cells, which contributes to epithelial dysfunction and mucus hypersecretion in COPD (94). This dual mode of action highlights the complexity of IL-33’s role in pulmonary diseases.

The IL-33/ST2 axis is also implicated in the regulation of immune responses beyond the lung. In autoimmune diseases, IL-33/ST2 signaling promotes pro-inflammatory cytokine release; while the specific mechanisms of immune cell recruitment remain unclear (91). IL-33 plays important roles in central nervous system homeostasis and disease mechanisms; however, evidence for protective, anti-inflammatory effects in neurodegenerative disorders remains inconclusive (87, 91). IL-33/ST2 signaling contributes to tissue repair and remodeling by modulating fibroblast activity and extracellular matrix production in chronic lung allograft dysfunction; evidence for liver fibrosis involvement is not supported (88, 96).

The axis also influences adaptive immunity by modulating T cell subsets. ST2 is expressed on a specialized subset of regulatory T cells (ST2+ Tregs) that respond to IL-33 by enhancing their suppressive functions, which can mitigate excessive inflammation and promote tissue repair (97, 98). Additionally, IL-33/ST2 signaling affects the expansion and function of memory Th2 cells, which are critical in sustaining T2 inflammation in chronic airway diseases (99). Notably, alternative promoter usage of the IL-33 receptor gene leads to differential ST2 expression in T cell subsets, influencing antiviral immune responses and inflammation (100).

The pathological implications of IL-33/ST2 signaling extend to infectious diseases and cancer. IL-33/ST2 axis protects against immunopathology in Trypanosoma cruzi infection by limiting inflammation and parasite burden; promotion of T2 immunity and contribution to disease pathology via excessive inflammation are not supported (101, 102). IL-33/ST2 signaling promotes glioma progression via tumor cell migration, invasion, and stemness; evidence for enhancing antitumor immunity or recruiting cytotoxic lymphocytes is not supported (88, 103).

In summary, the IL-33/ST2 signaling pathway serves as a critical mediator of immune activation and regulation, bridging innate and adaptive immunity. Its role as an alarmin enables rapid immune responses to tissue damage, while its broad cellular targets and signaling versatility contribute to both protective and pathological processes. Targeting the IL-33/ST2 axis holds promise for therapeutic interventions in COPD and inflammatory disorders; however, evidence for roles in autoimmunity and tumor immunity warrants further investigation (91, 93, 94). Further research is warranted to delineate context-specific functions and to develop strategies that modulate this pathway effectively without compromising host defense mechanisms.

### 5.2 Drug development targeting IL-33/ST2

The IL-33/ST2 axis has emerged as a critical pathway in the pathogenesis of COPD, representing a promising therapeutic target beyond traditional inhaled medications. IL-33, an alarmin cytokine belonging to the IL-1 family, is primarily released by epithelial and endothelial cells upon tissue damage or stress. IL-33 binds to its receptor ST2, which exists in membrane-bound and soluble forms; however, direct evidence for mediation of pro-inflammatory signaling contributing to airway inflammation, remodeling, and exacerbations in COPD is limited (93, 104). The development of biologics targeting IL-33 or ST2 aims to modulate these inflammatory cascades and potentially alter disease progression.

Tezepelumab, an anti- TSLP monoclonal antibody, indirectly influences the IL-33/ST2 axis by targeting upstream epithelial alarmins involved in T2 inflammation. While not directly targeting IL-33 or ST2, tezepelumab exemplifies the therapeutic potential of blocking epithelial-derived cytokines in COPD. Clinical trials have demonstrated that tezepelumab and other anti-alarmin agents, including itepekimab and tozorakimab (both anti-IL-33 antibodies), as well as astegolimab (an anti-ST2 antibody), show modest improvements in lung function and symptom control, although significant reductions in exacerbation rates remain inconsistent (34, 83). The modest efficacy observed may be partly attributable to the heterogeneous inflammatory endotypes within COPD populations, underscoring the importance of biomarker-guided patient selection to optimize therapeutic outcomes.

Astegolimab, a fully human IgG2 monoclonal antibody targeting ST2, has been evaluated in a phase 2a randomized, placebo-controlled trial (COPD-ST2OP) involving patients with moderate-to-very severe COPD. The study revealed that although astegolimab did not significantly reduce exacerbation rates compared to placebo, it improved health-related quality of life as measured by the Saint George’s Respiratory Questionnaire for COPD (SGRQ-C) and showed trends toward lung function improvement (84). Importantly, astegolimab demonstrated a favorable safety profile, with adverse event rates comparable to placebo and no increased risk of infections or major cardiac events, which is crucial given the immunomodulatory nature of IL-33/ST2 blockade (85). These findings suggest that targeting ST2 may confer symptomatic benefits and reduce eosinophilic inflammation, as reflected by significant reductions in blood and sputum eosinophil counts, although the impact on exacerbation prevention requires further investigation in larger, more targeted cohorts.

Preclinical and mechanistic studies have further elucidated the role of IL-33 in COPD pathophysiology. Notably, oxidized IL-33 (IL-33ox) can engage an alternative, ST2-independent signaling pathway via a receptor complex involving RAGE and EGFR on airway epithelial cells, leading to impaired epithelial repair and airway remodeling characteristic of COPD (94). This novel insight highlights the complexity of IL-33 biology and suggests that therapeutic strategies solely targeting the canonical IL-33/ST2 axis might not fully abrogate IL-33-driven pathogenic processes. Cigarette smoke exposure induces IL-33 release from lung fibroblasts, promoting tissue remodeling characterized by collagen deposition, which is a hallmark of COPD progression; evidence for release from other lung structural cells and fibrosis is limited (105). These observations imply that effective IL-33-targeted therapies may need to address both ST2-dependent and independent mechanisms to achieve comprehensive disease modification.

Clinical trials of anti-IL-33/ST2 biologics are ongoing, with several agents in phase II and III development aiming to clarify their efficacy in reducing exacerbations and improving lung function in biomarker-defined COPD subpopulations. The heterogeneity of COPD inflammatory phenotypes, including eosinophilic and non-eosinophilic endotypes, necessitates precision medicine approaches incorporating biomarkers such as blood eosinophil counts and IL-33/ST2 expression levels to identify responders (34, 83). Moreover, the IL-33/ST2 axis is implicated in the interplay between airway microbiota and autoimmune responses in COPD, as demonstrated by studies showing that microbial triggers like Edwardsiella tarda can induce IL-33-mediated autoantibody production and airway inflammation, further contributing to disease exacerbations and progression (24). This suggests that IL-33/ST2-targeted therapies might also modulate host-microbe interactions and immune dysregulation in COPD.

In summary, biologics targeting the IL-33/ST2 axis, such as tezepelumab and astegolimab, represent a novel class of therapeutic agents with potential to improve clinical outcomes in COPD by modulating airway inflammation, remodeling, and exacerbations. While current clinical trial data show promising safety and some efficacy signals, particularly in reducing eosinophilic inflammation and improving patient-reported outcomes, further large-scale, phenotype-stratified studies are essential to establish their definitive role in COPD management. Additionally, understanding the dual and context-dependent roles of IL-33, including ST2-independent pathways, will be critical for optimizing therapeutic strategies and developing next-generation agents with broader efficacy.

### 5.3 Clinical significance of IL-33/ST2 targeted therapy

The IL-33/ST2 axis has emerged as a pivotal mediator in the inflammatory processes underlying COPD influencing distinct inflammatory phenotypes and disease progression. IL-33, an alarmin cytokine primarily released by damaged airway epithelial cells, binds to its receptor ST2 to activate downstream inflammatory signaling pathways. This axis contributes to airway inflammation, remodeling, and immune cell activation, which are critical in COPD pathogenesis (106). Notably, the IL-33/ST2 pathway is involved in both T2 and non-T2 inflammatory responses, thereby affecting diverse COPD inflammatory endotypes. Elevated expression of IL-33 has been detected in airway epithelial cells from COPD patients; however, evidence for elevated ST2 expression in epithelial and immune cells and correlation with disease severity and progression is limited (94, 107). The presence of IL-33/ST2 signaling in non-hematopoietic lung cells such as epithelial, endothelial, and mesenchymal cells further amplifies local inflammation and remodeling. These findings underscore the role of IL-33/ST2 not only as a biomarker but also as a potential therapeutic target that modulates the inflammatory milieu characteristic of COPD.

The impact of IL-33/ST2 on COPD inflammatory subtypes is particularly relevant in the context of eosinophilic versus non-eosinophilic phenotypes. Studies have demonstrated increased numbers of innate lymphoid cells T2 expressing the ST2 receptor in eosinophilic COPD, suggesting that IL-33 signaling promotes eosinophilic inflammation via activation of ILC2s and subsequent IL-5 production (63). This mechanism may partly explain the heterogeneity in treatment responses observed in COPD patients, where those with higher eosinophilic inflammation may benefit more from therapies targeting IL-33/ST2. IL-33ox, acting via ST2-independent pathways, has been linked to airway epithelial remodeling, mucus hypersecretion, and impaired epithelial barrier function in COPD; direct involvement of IL-33/ST2 axis activation in these processes is not established (94, 105). The ST2 receptor exists in membrane-bound and soluble forms, with the latter acting as a decoy receptor that modulates IL-33 activity, adding further complexity to the regulation of this pathway in COPD. Collectively, these data highlight the IL-33/ST2 axis as a key driver of COPD inflammatory phenotypes and structural changes, providing a rationale for targeted intervention.

In terms of clinical outcomes, IL-33 and ST2 levels correlate inversely with pulmonary function parameters during acute exacerbations of COPD (AECOPD), indicating their potential role as biomarkers for disease severity and progression (108). Elevated serum concentrations of IL-33 and ST2 are associated with reduced FEV1% predicted and FEV1/FVC ratios, especially in older patients, males, and those with comorbid pulmonary hypertension, suggesting that IL-33/ST2 axis activity reflects exacerbation risk and lung function decline. Therapeutic targeting of this axis aims to mitigate these adverse outcomes. Clinical trials investigating astegolimab (anti-ST2) have shown mixed efficacy in reducing exacerbation rates and improvements in health status and eosinophil counts; while data on direct IL-33 antibodies (itepekimab, tozorakimab) are comparatively sparse (84, 85). For example, the phase 2a COPD-ST2OP trial with astegolimab indicated no statistically significant reduction in exacerbation rate compared to placebo but noted a clinically meaningful improvement in quality of life and a significant decrease in eosinophilic inflammation markers (84). These findings suggest that IL-33/ST2 blockade may confer benefits in symptom control and airway inflammation, even if exacerbation prevention remains modest.

Beyond inflammation, IL-33/ST2 signaling influences airway remodeling processes that contribute to irreversible airflow limitation in COPD. Experimental models have demonstrated that cigarette smoke exposure induces IL-33 translocation and release from lung fibroblasts, promoting collagen deposition and tissue remodeling via the IL-33/ST2 pathway (105). Furthermore, oxidized IL-33 can engage alternative receptors such as RAGE and EGFR independently of ST2, leading to impaired epithelial wound healing and increased mucus-producing cell differentiation, which exacerbate airway obstruction and muco-obstructive features characteristic of COPD (94). Neutralization of the IL-33ox pathway reversed these deleterious epithelial changes *in vitro*, suggesting that targeting IL-33 signaling may also protect lung structure and function. These mechanistic insights emphasize the multifaceted role of IL-33/ST2 in COPD pathogenesis, encompassing both inflammatory and remodeling pathways.

In summary, IL-33/ST2 targeted therapies hold promise for modifying COPD disease trajectory by addressing both inflammatory phenotypes and structural lung damage. While current clinical trial data indicate variable effects on exacerbation prevention, improvements in health status and reductions in eosinophilic inflammation support continued investigation of this pathway. The dual role of IL-33 in immune activation and tissue remodeling, including ST2-independent mechanisms, suggests that comprehensive blockade may be necessary for optimal clinical benefit. Future research should focus on patient stratification by inflammatory endotype, elucidation of IL-33/ST2 signaling complexity, and combination strategies to enhance lung function preservation and reduce acute exacerbations in COPD.

## 6 TSLP and the current research status of its inhibitors

### 6.1 TSLP in the immunoregulatory mechanism of COPD

TSLP is recognized as a pivotal upstream cytokine that orchestrates immune responses in chronic respiratory diseases, including COPD. As an epithelial-derived alarmin, TSLP is released rapidly upon airway epithelial injury caused by environmental insults such as cigarette smoke, pathogens, and pollutants. Its role as a master regulator of T2 immune responses is well established, primarily through its ability to activate dendritic cells (DCs), which subsequently promote the differentiation and activation of T helper 2 cells. This cascade leads to the amplification of T2 inflammation, which is characterized by eosinophilic infiltration, production of interleukins such as IL-4, IL-5, and IL-13, and enhanced airway hyperresponsiveness. In COPD, although traditionally regarded as a neutrophilic inflammation-driven disease, increasing evidence points to the presence of T2-high phenotypes in subsets of patients, especially those with eosinophilic inflammation (83). TSLP’s upstream positioning allows it to modulate both T2-high and T2-low immune responses, making it a critical mediator in the complex inflammatory milieu of COPD.

TSLP’s interaction with dendritic cells is crucial for initiating and sustaining airway inflammation. Upon release, TSLP binds to its heterodimeric receptor complex composed of the **TSLP** receptor (TSLPR) and IL-7 receptor alpha (IL-7Rα) on DCs. This binding induces DC maturation and upregulation of co-stimulatory molecules, facilitating the priming of naïve T cells towards a Th2 phenotype. The activated Th2 cells then secrete cytokines that promote eosinophil recruitment and mucus production in the airway epithelium. This mechanism contributes to airway remodeling and persistent inflammation characteristic of COPD exacerbations. TSLP is detectable in airway epithelial cells of COPD patients; however, evidence for increased expression during exacerbations and correlation with enhanced inflammatory mediator secretion is not established (25). This suggests that TSLP not only acts as an initiator but also perpetuates airway inflammation and structural changes.

Beyond its role in cellular activation, TSLP directly influences airway epithelial function by promoting mucus hypersecretion, a hallmark of COPD pathology. The overproduction of mucus contributes to airway obstruction and impaired mucociliary clearance, exacerbating disease progression. Studies have demonstrated that TSLP can upregulate mucin gene expression in epithelial cells, thereby increasing mucus production. This effect is often accompanied by the recruitment of eosinophils and other inflammatory cells, further amplifying the inflammatory response. TSLP and HMGB1 are key mediators of airway inflammation; although the potential synergistic interplay between TSLP, IL-33, and IL-25 remains a subject for future research (109). The cumulative effect of these interactions underscores TSLP’s central role in modulating both immune cell activation and epithelial barrier dysfunction in COPD.

Clinical observations reinforce the immunoregulatory importance of TSLP in COPD. Patients with elevated serum TSLP levels tend to exhibit more severe disease phenotypes, including increased eosinophilic inflammation, higher exacerbation rates, and greater airflow limitation. Although much of the detailed characterization has been derived from asthma cohorts, parallels in COPD suggest a subset of patients with a TSLP-driven inflammatory endotype. This subgroup may particularly benefit from targeted therapies against TSLP. The development of tezepelumab, a human monoclonal antibody that blocks TSLP-TSLPR interaction, has shown promise in clinical trials for asthma and is currently under investigation for COPD treatment (110). These therapeutic advances highlight the potential of TSLP blockade to modulate upstream inflammatory pathways, thereby reducing exacerbations and improving clinical outcomes in COPD.

In summary, TSLP serves as a crucial upstream cytokine in COPD immunoregulation by activating dendritic cells and Th2 lymphocytes, promoting airway inflammation, and enhancing mucus secretion. Its role bridges innate and adaptive immunity, contributing to the heterogeneity of COPD phenotypes. The targeting of TSLP and its signaling pathways represents a promising therapeutic avenue that could complement existing inhaled treatments, particularly for patients with eosinophilic or T2-high COPD profiles. Further studies are warranted to delineate the full spectrum of TSLP’s actions in COPD and to optimize the clinical application of anti-TSLP therapies.

### 6.2 TSLP-targeted drugs and their mechanisms of action

TSLP has emerged as a critical epithelial-derived cytokine involved in the initiation and amplification of airway inflammation, making it a promising therapeutic target in chronic inflammatory airway diseases such as asthma and COPD. Tezepelumab, a first-in-class human IgG2λ monoclonal antibody targeting TSLP, has been the pioneer in this field, receiving approval for severe asthma treatment. It functions by binding to TSLP and preventing its interaction with the TSLP receptor complex, thereby inhibiting downstream inflammatory signaling cascades involving dendritic cells and innate lymphoid cells; involvement of T cells and B cells is not established (111, 112). Clinical studies indicate that tezepelumab demonstrates broad efficacy across various asthma phenotypes, highlighting its potential role in targeting upstream airway inflammation (113, 114). Moreover, tezepelumab’s approval marks a significant advancement, as it is the only biologic currently indicated in severe asthma without restrictions based on phenotype or biomarker status, reflecting its wide therapeutic potential (111).

Beyond systemic administration, innovative inhaled formulations targeting TSLP are under development to enhance local drug delivery to the lungs while minimizing systemic exposure and related adverse effects. Ecleralimab (CSJ117), an inhaled antibody fragment against soluble TSLP, exemplifies this approach by directly neutralizing TSLP in the airway lumen, thereby preventing receptor activation and subsequent inflammatory signaling (112). Preclinical and early-phase clinical studies have demonstrated that ecleralimab reduces allergen-induced bronchoconstriction and is well tolerated in mild asthma patients, suggesting its potential as a novel therapeutic class (112). Similarly, LQ043, a bivalent inhalable nanobody targeting TSLP, has shown promising preclinical efficacy in murine models of allergic airway inflammation and favorable pharmacokinetic and safety profiles in nonhuman primates and phase I clinical trials (115). The inhaled delivery of such biologics offers advantages in terms of targeted lung tissue penetration and potentially lower treatment costs due to scalable production techniques such as yeast expression systems (115). These developments indicate a future trend toward more localized and patient-friendly TSLP-targeted therapies.

In addition to monoclonal antibodies and nanobodies, several small molecules and natural compounds have demonstrated regulatory effects on TSLP expression, expanding the therapeutic landscape. Arctigenin, a bioactive lignan from Arctium lappa, has been shown to downregulate TSLP expression via NF-κB pathway modulation in breast cancer cells; While Arctigenin shows promise, the effects of other compounds like EGCG remain to be characterized in this specific context (116). Arctigenin decreases TSLP expression and other tumor-promoting cytokines in breast cancer models; however, its potential in modulating inflammatory pathways beyond airway diseases is not established (116). These natural products may exert anti-inflammatory effects by inhibiting the transcriptional activation of TSLP, thereby attenuating downstream STAT3 and β-catenin signaling involved in cell proliferation and inflammation (116). Such findings suggest that adjunctive therapies based on natural compounds could complement biologic treatments by targeting TSLP at the gene expression level, potentially offering cost-effective and accessible options for chronic inflammatory diseases.

Emerging research also highlights the interplay between TSLP and other cytokine signaling pathways, suggesting that combination therapies targeting multiple upstream mediators may enhance therapeutic efficacy. For example, the novel humanized anti-TSLP monoclonal antibody QX008N demonstrated superior *in vitro* efficacy in blocking TSLP-induced signaling compared to tezepelumab and showed enhanced anti-inflammatory effects when combined with anti-IL-4 receptor antibodies in preclinical models of asthma (117). This dual targeting approach addresses the complex cytokine milieu in airway inflammation, potentially overcoming limitations of monotherapy by simultaneously inhibiting parallel pathways involved in **T2** immune responses. Such strategies could be particularly beneficial for patients with severe or refractory disease phenotypes where multiple inflammatory axes are active.

In COPD, TSLP is recognized as a key alarmin released by airway epithelial cells in response to environmental insults such as cigarette smoke; however, clinical evidence for anti-TSLP therapies is currently lacking (81, 118). TSLP is an upstream regulator of T2-high and T2-low immune responses; however, correlations between TSLP levels and disease severity or promotion of diverse inflammatory responses are not established (83). Inhalable TSLP-targeted nanobodies like LQ043 show promise in modulating airway epithelial inflammation with reduced systemic exposure in asthma models, although data regarding its application in COPD patients are currently sparse (115). However, further clinical trials are needed to confirm efficacy and safety in this population.

Collectively, the landscape of TSLP-targeted therapeutics is rapidly evolving, encompassing approved monoclonal antibodies like tezepelumab, innovative inhaled biologics such as ecleralimab and nanobodies, as well as small molecules and natural products that modulate TSLP expression. These advances reflect a growing understanding of TSLP’s central role in airway inflammation and underscore the potential for precision medicine approaches that tailor therapies based on patient-specific inflammatory profiles. Continued research into combination therapies and novel delivery systems promises to expand the clinical utility of TSLP inhibition in asthma, COPD, and potentially other chronic inflammatory diseases.

### 6.3 Clinical studies and therapeutic prospects

The therapeutic potential of TSLP inhibitors in COPD has garnered significant attention, particularly regarding their role in managing acute exacerbations and controlling airway inflammation. TSLP, an epithelial-derived alarmin cytokine, acts upstream in the inflammatory cascade, orchestrating T2 immune responses that contribute to airway remodeling and exacerbation risk. Clinical trials investigating tezepelumab, a monoclonal antibody targeting TSLP, have shown promising results in reducing exacerbation rates and improving lung function in COPD patients with elevated eosinophil counts. Although modest FEV1 improvements have been reported in eosinophilic subgroups, consistent reductions in annualized exacerbation rates with TSLP blockade are not clearly demonstrated (35, 54). TSLP inhibition may modulate airway inflammation in COPD; however, direct evidence for effects on airway remodeling and mucus hypersecretion is limited (58).

The integration of TSLP inhibitors into COPD management paradigms is further supported by their favorable safety profiles observed in randomized controlled trials. Tezepelumab, itepekimab, and astegolimab have been investigated in COPD; however, the applicability of severe asthma evidence to COPD remains to be validated (34). However, the clinical efficacy of these biologics in non-eosinophilic COPD phenotypes remains less clear, with some studies reporting non-significant effects on exacerbation reduction and lung function improvement in broader COPD cohorts. This underscores the importance of biomarker-driven patient selection to optimize therapeutic outcomes and minimize unnecessary exposure (34). Biologics overall have been associated with reduced systemic glucocorticoid usage in COPD; however, specific evidence for TSLP inhibitors in this regard is lacking (119).

Exploring combination therapies involving TSLP inhibitors and other biologics targeting complementary inflammatory pathways presents a compelling avenue to enhance treatment efficacy in COPD. Given the heterogeneity of COPD inflammation, characterized by overlapping T2 and non- T2 endotypes, co-targeting upstream alarmins like TSLP and IL-33 or downstream effectors such as IL-5 and IL-4/IL-13 receptors may provide synergistic benefits. Combining TSLP blockade with anti-IL-5 therapies is a potential strategy to target multiple inflammatory pathways in COPD; however, evidence for enhanced clinical benefits is not established (58). Furthermore, dual targeting strategies may also attenuate airway remodeling processes more effectively than monotherapy, as airway structural changes are driven by multiple interconnected cytokine networks. Clinical data on combination biologic regimens in COPD are limited, although the direct applicability of severe asthma evidence to COPD is not yet fully established (120).

Another promising therapeutic prospect involves the sequential or concomitant use of TSLP inhibitors with inhaled therapies, including corticosteroids and long-acting bronchodilators. This integrated approach could optimize control of airway inflammation and obstruction, particularly in patients exhibiting frequent exacerbations despite maximal inhaled therapy. Real-world prescribing trends show increased use of biologics and inhaled therapies in patients with overlapping asthma-COPD features; however, clinical recognition of personalized strategies remains a challenge (121). Moreover, the potential of TSLP inhibitors to reduce exacerbation-related hospitalizations and improve quality of life measures, such as St. George’s Respiratory Questionnaire scores, further supports their incorporation into comprehensive COPD management plans (54).

In summary, TSLP inhibitors represent a promising class of biologics with the potential to address unmet needs in COPD, particularly in patients with T2 inflammatory signatures. Their role in reducing acute exacerbations, improving symptom control, and decreasing systemic corticosteroid burden positions them as valuable additions to the therapeutic armamentarium. The exploration of combination biologic therapies and integration with existing inhaled treatments could further enhance clinical benefits. Nonetheless, ongoing and future large-scale, biomarker-stratified clinical trials are essential to delineate optimal patient selection criteria, evaluate long-term safety and efficacy, and clarify the potential of these agents to modify disease progression in COPD.

## 7 Other related biologics and combination therapy strategies

### 7.1 JKN2401, and other novel biologics in development

The development of novel biologics such as JKN2401 and TQC2731 represents a promising frontier in the management of COPD, particularly for patients who remain symptomatic despite optimal inhaled therapies. These biologics primarily target upstream inflammatory mediators implicated in COPD pathogenesis, including TSLP and related alarmins, which orchestrate complex immune responses contributing to airway inflammation and remodeling.

JKN2401 is a monoclonal antibody designed to antagonize the TSLP receptor, thereby inhibiting TSLP-mediated activation of downstream inflammatory pathways. This targeted approach aims to modulate both T2 and non-T2 inflammation, addressing the heterogeneity observed in COPD patients. Early-phase clinical trials have demonstrated that JKN2401 exhibits high *in vitro* bioactivity against TSLP receptor signaling, with a favorable safety profile. Although data remain preliminary, the mechanism of action suggests potential benefits in reducing exacerbations and improving lung function by attenuating epithelial alarmin-driven inflammation (86).

Similarly, TQC2731 is a monoclonal antibody with a high affinity for TSLP itself, effectively neutralizing this cytokine before it engages its receptor. Preclinical studies indicate that TQC2731 possesses potent inhibitory effects on TSLP activity, which may translate into clinical efficacy by suppressing airway inflammation and mucus hypersecretion. The pharmacological properties of TQC2731, including its pharmacokinetics and immunogenicity, are under ongoing investigation. TQC2731 shows high *in vitro* bioactivity as a TSLP inhibitor, and its specific therapeutic potential in distinct COPD phenotypes is currently under evaluation (86).

Beyond these agents, other biologics targeting related pathways are under development, including inhaled antibody fragments such as ecleralimab, which may provide enhanced tissue penetration with reduced systemic exposure, potentially minimizing adverse effects. Bispecific antibodies like SAR443765, which simultaneously inhibit TSLP and interleukin-13 (IL-13), represent an innovative strategy to interfere with multiple inflammatory axes concurrently, potentially offering superior efficacy in complex inflammatory endotypes (86).

Importantly, these novel biologics may demonstrate synergistic effects when combined with traditional COPD treatments such as long-acting bronchodilators and inhaled corticosteroids. For instance, targeting upstream cytokines like TSLP may complement bronchodilator-induced airway smooth muscle relaxation by reducing the underlying inflammation that perpetuates airway obstruction and remodeling. Combination therapies may reduce exacerbations in COPD patients with specific inflammatory profiles; however, evidence for optimization of symptom control and quality of life improvements remains limited (122).

While the initial clinical data are encouraging, several challenges remain. The heterogeneity of COPD necessitates precise patient stratification, often guided by biomarkers such as blood eosinophil counts or alarmin expression levels, to identify those most likely to benefit from these therapies. Furthermore, long-term safety and efficacy data are required to establish the role of these agents in routine clinical practice. Cost considerations and accessibility will also influence their adoption, underscoring the need for ongoing research and health economic evaluations (86).

In summary, the development of biologics such as JKN2401 and TQC2731 marks a significant advancement in COPD therapeutics, targeting key upstream inflammatory mediators. Their integration with existing therapies holds promise for more personalized and effective management strategies, particularly for patients with T2-high or alarmin-driven COPD phenotypes. Continued clinical investigation will clarify their therapeutic potential and guide their optimal use in combination regimens.

### 7.2 Targeting multi-pathway combination therapeutic strategies

The complexity and heterogeneity of COPD pathogenesis, especially in the context of T2 inflammation, necessitate innovative therapeutic approaches that target multiple inflammatory pathways simultaneously. Biologic agents targeting IL-4 receptor, IL-5 receptor, IL-33, and TSLP have individually shown promising efficacy in subsets of COPD patients, particularly those with eosinophilic phenotypes. Given their distinct yet overlapping roles in airway inflammation and immune regulation, combination therapies that simultaneously inhibit these pathways hold significant potential to enhance clinical outcomes beyond monotherapy.

IL-4Rα blockade, exemplified by dupilumab, inhibits both IL-4 and IL-13 signaling, leading to broad suppression of T2 cytokine-driven inflammation. Clinical trials demonstrate that dupilumab significantly reduces exacerbation rates and improves lung function in COPD patients with elevated eosinophils; the impact on quality of life requires further confirmation (34, 54). Meanwhile, targeting IL-5 or IL-5Rα with agents such as mepolizumab and benralizumab has shown moderate efficacy in reducing exacerbations in eosinophilic COPD, although these effects are less robust compared to IL-4R blockade (34, 54). IL-33 and its receptor ST2 are upstream epithelial-derived alarmins that activate innate lymphoid cells Th2 and other immune cells, amplifying airway inflammation. Biologics such as itepekimab, astegolimab, and tozorakimab have demonstrated mixed results with modest lung function gains but largely non-significant effects on exacerbation rates in COPD patients (34, 58). TSLP, another epithelial alarmin, orchestrates both T2-high and T2-low inflammatory responses, making it an attractive target. Tezepelumab, an anti-TSLP antibody, is under active investigation; however, clinical outcomes in COPD show limited or inconsistent benefits (34, 58).

The rationale for combining these biologics lies in their complementary mechanisms of action. While IL-4R and IL-5/IL-5R blockade primarily target adaptive immune responses and eosinophilic inflammation, IL-33/ST2 and TSLP inhibitors act upstream, modulating epithelial-immune cell crosstalk and innate immunity. This multi-level inhibition could theoretically provide more comprehensive control of airway inflammation, addressing both eosinophilic and non-eosinophilic pathways that contribute to COPD pathophysiology. Concurrent targeting of multiple inflammatory pathways is a proposed strategy; however, evidence for mitigation of compensatory upregulation with single-pathway blockade is not established (34, 58). However, the safety and pharmacokinetic profiles of such combinations require careful evaluation to avoid additive immunosuppression or unforeseen adverse effects.

Personalized treatment design is critical when considering multi-pathway combination strategies. Biomarker-guided approaches using blood eosinophil counts and sputum markers can stratify patients for ICS responsiveness; evidence for FeNO and biologic therapy stratification is limited (57, 123). For example, patients with high eosinophil counts and elevated IL-4/IL-13 signaling may respond best to IL-4R antagonists, whereas those with increased epithelial alarmin expression might derive more benefit from IL-33 or TSLP blockade. Integration of molecular phenotyping and clinical characteristics can refine patient selection and optimize therapeutic efficacy while minimizing unnecessary exposure to costly biologics (57, 58). Sex- and age-related differences in COPD progression have been observed; however, evidence for differences in immune responses and drug metabolism affecting individualized pharmacotherapy is limited (124, 125).

Current evidence also suggests that combining biologics with inhaled corticosteroids or long-acting bronchodilators may further enhance therapeutic outcomes. ICS use can modulate airway microbiome and metabolite profiles; however, effects on inflammatory milieu and responsiveness to biologics are not established (126, 127). The integration of biologics with optimized inhaled therapies and non-pharmacological interventions such as pulmonary rehabilitation may provide a holistic, multi-modal approach to COPD management. Emerging nanotechnology-based delivery systems and exosome-mediated therapeutics offer innovative platforms to enhance targeted delivery and reduce systemic exposure; evidence for enabling combination drug delivery with controlled release kinetics is limited (128, 129).

In summary, targeting multiple inflammatory pathways through combination biologic therapies holds promise for advancing COPD treatment, particularly in patients with complex, overlapping inflammatory endotypes. The success of such strategies hinges on precise patient phenotyping, biomarker-driven selection, and careful balancing of efficacy and safety. Ongoing and future clinical trials and real-world evidence will be important in defining the role of biologics in personalized COPD care; however, specific combination trials of IL-4R, IL-5R, IL-33, and TSLP inhibitors have not yet been widely reported (34, 54).

### 7.3 Immunomodulators and small molecule drugs as adjunctive therapies

Phosphodiesterase-4 (PDE4) inhibitors, such as roflumilast, represent a class of small molecule drugs that exert significant immunomodulatory effects in COPD. PDE4 is a key enzyme responsible for degrading cyclic adenosine monophosphate (cAMP) in inflammatory cells, and its inhibition leads to increased intracellular cAMP levels, which subsequently suppress the release of pro-inflammatory cytokines such as tumor necrosis factor-alpha (TNF-α), interleukin-6 (IL-6), and chemokines involved in neutrophil recruitment. Roflumilast has been shown to reduce airway inflammation, improve lung function, and decrease the frequency of exacerbations in COPD patients, particularly those with chronic bronchitis phenotypes. The immunoregulatory mechanism of PI3Kδ inhibitors involves modulation of multiple immune cells, including neutrophils, macrophages, and T lymphocytes, dampening excessive inflammatory responses that contribute to airway remodeling and disease progression, but the direct impact of PDE4 inhibitors on this specific pathway requires further verification (130). Additionally, PDE4 inhibitors may enhance antiviral immunity by promoting interferon responses, as evidenced by studies where selective PI3Kδ inhibition, which shares overlapping signaling pathways with PDE4, improved antiviral interferon production and reduced viral replication in airway epithelial cells. This suggests that PDE4 inhibitors might also indirectly influence antiviral defense mechanisms in COPD, potentially mitigating viral-induced exacerbations.

Natural products have gained increasing attention for their anti-inflammatory and immunomodulatory properties in COPD management. Various phytochemicals derived from plants, such as flavonoids, alkaloids, and terpenoids, have demonstrated the ability to interfere with key inflammatory signaling pathways, including nuclear factor-kappa B (NF-κB), mitogen-activated protein kinases (MAPKs), and the NLRP3 inflammasome. These compounds can reduce oxidative stress and inhibit the release of pro-inflammatory mediators, thereby attenuating chronic airway inflammation. For example, some natural antioxidants can scavenge reactive oxygen species (ROS), which are elevated in COPD and contribute to tissue damage and inflammation. Moreover, certain natural products modulate immune cell function, promoting a shift from a pro-inflammatory to a regulatory phenotype, which may help restore immune homeostasis in the lungs. The integration of natural products as adjunctive agents could complement conventional therapies by targeting multiple inflammatory pathways simultaneously, providing a broader anti-inflammatory effect with potentially fewer side effects.

Beyond their direct anti-inflammatory actions, immunomodulators and natural products may also influence systemic inflammation and comorbidities associated with COPD. Chronic inflammation in COPD is linked to cardiovascular diseases, diabetes, and other systemic conditions, partly mediated by circulating inflammatory mediators and immune complexes. Targeting systemic immune dysregulation through PDE4 inhibition or natural compounds might reduce the burden of these comorbidities. Elevated levels of autoantibodies against advanced glycation end products (AGEs) have been observed in coronary artery disease patients, with higher prevalence of COPD as a comorbidity; immune mechanisms may contribute to systemic pathology (131). Modulating immune responses with PDE4 inhibitors or natural products could potentially mitigate such autoimmune-like processes, though further research is needed to clarify these effects.

In addition, emerging evidence suggests that immunomodulatory therapies might impact the lung microenvironment’s antiviral defenses, which is crucial given the role of viral infections in exacerbating COPD. Studies have shown that targeting pathways such as PI3Kδ can enhance antiviral interferon responses and reduce viral replication in airway epithelial cells, thereby preventing prolonged inflammation and tissue damage (130). Since PDE4 inhibitors also affect similar intracellular signaling cascades, they may share this beneficial antiviral property, contributing to reduced exacerbation frequency. This dual anti-inflammatory and antiviral action positions immunomodulators as promising adjunctive treatments in comprehensive COPD management.

Taken together, PDE4 inhibitors and natural products offer valuable immunomodulatory effects that extend beyond bronchodilation and symptom control in COPD. Their ability to attenuate airway and systemic inflammation, modulate immune cell function, and potentially enhance antiviral defenses supports their adjunctive use alongside targeted biologics. Future studies should focus on elucidating the precise molecular mechanisms, optimizing combination therapies, and evaluating clinical outcomes to fully harness these agents’ therapeutic potential.

## 8 Conclusion

The therapeutic landscape of COPD is shifting from broad-spectrum symptom control towards precision medicine, driven by the recognition of distinct inflammatory endotypes. Biologics targeting the IL-4R, IL-5/IL-5R, IL-33/ST2, and TSLP pathways have emerged as promising interventions to reduce exacerbations and improve lung function, demonstrating clear efficacy in patients with eosinophilic or type 2-high signatures. However, the complexity of COPD pathogenesis, particularly involving non-type 2 inflammation and upstream alarmin pathways, necessitates better patient stratification beyond simple blood eosinophil counts. Current challenges, including the identification of robust companion biomarkers and the optimization of treatment timing, must be addressed through rigorous translational research. Moving forward, the integration of multi-dimensional biomarker profiling and the exploration of combination biologic strategies will be essential to overcome disease heterogeneity and fully realize the potential of personalized COPD management.
